# Social autopsy for maternal and child deaths: a comprehensive literature review to examine the concept and the development of the method

**DOI:** 10.1186/1478-7954-9-45

**Published:** 2011-08-05

**Authors:** Henry D Kalter, Rene Salgado, Marzio Babille, Alain K Koffi, Robert E Black

**Affiliations:** 1 Department of International Health, Johns Hopkins Bloomberg School of Public Health, (615 North Wolfe Street), Baltimore, (21205), USA; 2 President's Malaria Initiative, United States Agency for International Development, (1300 Pennsylvania Avenue), Washington, DC, (20523), USA; 3 UNICEF, Chad country office, (PO Box 1146), N'Djamena, Chad

## Abstract

"Social autopsy" refers to an interview process aimed at identifying social, behavioral, and health systems contributors to maternal and child deaths. It is often combined with a verbal autopsy interview to establish the biological cause of death. Two complementary purposes of social autopsy include providing population-level data to health care programmers and policymakers to utilize in developing more effective strategies for delivering maternal and child health care technologies, and increasing awareness of maternal and child death as preventable problems in order to empower communities to participate and engage health programs to increase their responsiveness and accountability.

Through a comprehensive review of the literature, this paper examines the concept and development of social autopsy, focusing on the contributions of the Pathway Analysis format for child deaths and the Maternal and Perinatal Death Inquiry and Response program in India to social autopsy's success in meeting key objectives. The Pathway Analysis social autopsy format, based on the Pathway to Survival model designed to support the Integrated Management of Childhood Illness approach, was developed from 1995 to 2001 and has been utilized in studies in Asia, Africa, and Latin America. Adoption of the Pathway model has enriched the data gathered on care seeking for child illnesses and supported the development of demand- and supply-side interventions. The instrument has recently been updated to improve the assessment of neonatal deaths and is soon to be utilized in large-scale population-representative verbal/social autopsy studies in several African countries. Maternal death audit, starting with confidential inquiries into maternal deaths in Britain more than 50 years ago, is a long-accepted strategy for reducing maternal mortality. More recently, maternal social autopsy studies that supported health programming have been conducted in several developing countries. From 2005 to 2009, 10 high-mortality states in India conducted community-based maternal verbal/social autopsies with participatory data sharing with communities and health programs that resulted in the implementation of numerous data-driven maternal health interventions.

Social autopsy is a powerful tool with the demonstrated ability to raise awareness, provide evidence in the form of actionable data and increase motivation at all levels to take appropriate and effective actions. Further development of the methodology along with standardized instruments and supporting tools are needed to promote its wide-scale adoption and use.

## Introduction and background

In developing country settings with inadequate vital registration systems and where many deaths occur at home, verbal autopsy is the investigative method most often used to determine the prevailing biological causes of death. Health policymakers and programmers require these data to identify health priorities, allocate sparse resources, and evaluate the impact of health programs. Social autopsy consists of questions on modifiable social, cultural, and health system factors that contribute to the same deaths investigated by verbal autopsy. Because social autopsy studies are often conducted without a control group of survivors, it is important that the factors included be based on interventions of proven efficacy. Health care programmers and policymakers need these data to identify strategies for increasing health-promotive behaviors and access to and utilization of quality health care services. Two complementary purposes of social autopsy are to increase awareness of maternal and child mortality to empower communities to participate and to engage health programs to increase responsiveness and accountability; and to provide large-scale population-level data to support advocacy and securing of the necessary resources to tackle these problems.

Verbal autopsy instruments for child deaths have most often included only limited elements that could be termed "social autopsy," usually consisting of a few questions regarding whether and where care was sought for the fatal illness. In contrast, verbal autopsies for maternal deaths earlier on and more frequently have examined the social contributors to death alongside the medical causes. Factors influencing this approach include the success of the nationwide system of health facility-based confidential inquiry into maternal deaths conducted in the United Kingdom since 1952, which from the beginning recognized the importance of social factors and examined these by constructing illustrative vignettes of individual maternal deaths [1]; and later, the widespread adoption of the "Three Delays" model of maternal mortality [2], which highlights the social/behavioral causal chain linking the household, community, and health system and provides a clear framework for the development of maternal social autopsy tools. The World Health Organization (WHO) helped promote the spread of maternal death reviews using several methods, including verbal autopsy with a strong social element, with its Beyond the Numbers effort [3], which was highly influenced by the earlier work in Britain.

Similarly influenced by Mosley and Chen's 1984 framework for the study of child survival in developing countries, which posited a set of socioeconomic determinants underlying and operating through proximate biological factors to affect mortality [4], child survival strategies in the 1990s evolved in the same direction toward integrated approaches and an appreciation for the importance of household and community factors in health promotion, disease prevention, and treatment. This culminated in the development of the Pathway to Survival framework in 1995 [5]. In response, social autopsy efforts for fatally ill children emerged that holistically track the entire process and determinants of health care provision, care seeking (or not) from home to facility, and the quality of care provided.

The size and scope of the enhanced social autopsy efforts both for child and maternal deaths varied, but most were limited to studies at subdistrict, district, or country-region level. This was often appropriate, as important social factors may vary by site and many social autopsy studies were intended to support local health program implementation. But national level data, important for advocating and securing resources for community health approaches, as well as for developing regional and global estimates of social and behavioral determinants of health, were lacking. An anomaly emerged as well -- that, while the methodology grew from a programmatic approach that acknowledges the importance of community participation, few of the programs or researchers conducting social autopsies have sought participation below the level of health programmers and policymakers in sharing or utilizing the data for program or intervention development. However, a track did emerge among practitioners and external users of maternal death reviews, including those based on verbal autopsy, recognizing the power of the data to increase the visibility and awareness of the problem [6] and, in the process, to raise the demand for access to quality maternal health care as a human right [7]. In this way, social autopsy has made an important contribution to the political process and formation of health policy at the global, national, and subnational levels.

This paper undertakes a comprehensive review of the literature in order to examine the concept of social autopsy, the development of the methodology, and the quality of its execution in the pursuit of five key objectives. The analysis of study outcomes is organized around seminal efforts in which the authors have participated, while also assessing how widely and successfully the social autopsy method has been adopted.

## Methods

### Search strategy

We conducted computerized searches of Embase, PubMed, and SCOPUS databases using the keywords and phrases: (careseeking OR care-seeking OR care seeking) AND (death OR mortality), "social autopsy," and "verbal autopsy." We then manually searched references quoted in original publications for additional information.

### Study inclusion and exclusion criteria

To be included in the review, studies had to fulfill the following criteria:

1. Published after 1989 in a peer-reviewed journal or as a report accessible through a Web search;

2. Examine the care-seeking process for fatal illnesses of children from birth to 5 years or for maternal deaths;

3. Investigate a minimum of 50 child or maternal deaths;

4. Include an abstract accessible through the search database; and

5. Written in English or French.

### Study characteristics

Standard information was abstracted from all eligible studies by two reviewers (AK and HDK). The information included the following: the dates the data were collected and published; the setting, i.e., the country and site in which the work was conducted; the group studied (maternal, child, or both); the number of deaths observed; the study objective and design; and the format (open-ended, closed-ended, or combined) and source of the social autopsy questionnaire.

### Outcomes

Data were also extracted to assess whether the study met five key objectives of social autopsy, as follows: *(i) essential elements of the care-seeking process *were described, including recognition of the illness, whether adequate home care was provided, whether and what type of outside-the-home care was sought (informal, formal, or both), delays to formal health care seeking and related constraints (e.g., lack of knowledge of illness danger signs, seeking traditional care, lack of transportation, costs), and the quality of health care provided (from the client's perspective); *(ii) a social diagnosis of the contributors to death *was made, i.e., household (behavioral), community (social), and health system determinants of the deaths were identified; *(iii) the study provided representative national or large area data; *and the data were utilized to support *(iv) health program or policy development; *and/or *(v) community empowerment*.

### Ethical considerations

The social autopsy studies conducted in Bolivia [8] and Guinea [9] that two of the authors participated in and that were central to the work described in this paper were programmatic efforts approved by the national and regional ministries of health (MOH) of the respective countries without undergoing formal ethical review. The Maternal and Perinatal Death Inquiry and Response (MAPEDIR) program in India [10], also central to this paper, was reviewed by the Johns Hopkins University institutional review board and found to be a programmatic effort, rather than research, and so was exempt from board oversight. Nevertheless, key Helsinki principles were upheld in the conduct of all these studies, including administering informed consent to all respondents and maintaining the confidentiality of the information they provided.

## Results

The search of the databases using the keywords and phrases identified 14 articles and reports of child deaths and eight of maternal deaths that met the inclusion criteria (Table 1). Only three child studies were conducted prior to the development of the Pathway to Survival model in 1995, which positively influenced the scope of care-seeking factors considered by subsequent studies. Understanding the Pathway sets the context for examining the development of the social autopsy method.

**Table 1:** Studies and reports meeting the inclusion criteria of the comprehensive review

Study		Study characteristics			Outcomes				
**Author and reference #**	**Publication date**	**Study setting**	**Age group studied**	**No. of deaths investi-gated**	**Data collected on the care-seeking process: 1) illness recognition; 2) home care; 3) recognition of severe illness; 4a) times, 4b) sequence, and 4c) type of health care sought; 5) CS delays; 6) CS constraints; 7) quality of care; 8) referral; 9) compliance with home care and/or referral advice**	**Social diagnosis of contributors to death was made**	**Data provided** **were: 1)** **representative;** **2) large area** **(district/** **regional/** **national)**	**Data were utilized to** **support health** **program or policy** **development: 1)** **advocacy/** **accountability; 2)** **data sharing and** **interpretation; 3)** **intervention** **development =** **responsiveness**	**Data were utilized to support community empowerment: 1) data sharing and interpretation; 2) intervention development; 3) monitoring and revision**

Sustrisna [13]	1993	Indonesia: 10,000 HHs, Indramayu, West Java	Under 5 years old	139	4c; 6	Implied	1) Unclear; 2) No	1/2/3) None stated	1/2/3) None stated

Gutierrez [14]	1994	Mexico: Tlaxcala state	3 days-5 years old	98 ARI & 34 acute diarrhea	4c, 5; 7; 8	Implied	1) Unclear 2) Yes	□1/2/3) Yes	1/2/3) None stated

Sodemann [15]	1997	Guinea-Bissau: 2 suburbs of Bissau	1-30 months old	125	4a, c; 5; 7	Yes	1) Yes; 2) No	1/2/3) None stated	1/2/3) None stated

Aguilar [8]	1998	Bolivia: El Alto city	Under 5 years old	271	PtoS study: 1; 2; 3; 4b, 4c; 5; 7; 9	Yes	1) Likely; 2) No	△1) Yes; 2/3) None stated	△1) Yes; 2/3) None stated

de Bocaletti [19]	1999	Guatemala: 4 towns	Stillbirths & 0-6 days old	101/36	PtoS study: A) Mother: delivery place & decision maker; B) Mother & child: 1; 2; 3; 4c; 5; 6; 7; 9	Yes	1) Likely; 2) No	**□**1/2) Yes; 3) Goals stated	**□**1/2) Yes; 3) None stated

de Souza [20]	2000	Brazil: 11 municipalities, Ceara state	1-11 months old	127	PtoS study: 2; 3; 4a, b, c; 5; 6; 7; 9	Yes	1) Possible; 2) Yes	1/2/3) None stated	△1) Yes; 2/3) None stated

RACHA [21]	2000	Cambodia: 40 villages in 4 provinces	Perinates & 1 wk.-59 mo. Old	59/119	PtoS study: A) Mother: delivery place & decision maker; B) Mother & child: 1; 2; 3; 4a, b, c; 5; 6; 7; 9	Yes	1) Possible; 2) Yes	**□**1/2) Yes; 3) Goals stated	1) None stated; 2) Goal to mobilize the community; 3) None stated

Bhandari [22]	2002	India: 2 urban slums, Delhi	0-365 days	162	PtoS study: 3; 4a, b, c; 5; 7; 9; referral compliance constraints	Yes	1) No; 2) No	1/2/3) None stated	1/2/3) None stated

Schumacher [9]	2002	Guinea: Mandiana prefecture	0 days-59 months old	330	PtoS study: 1; 2; 3; 4a, b, c; 5; 6; 7; 9	Yes	1) Yes; 2) Yes	**□**1/2/3) Yes	**□**1/2) Yes; 3) None stated

Hinderaker [16]	2003	Tanzania: 2 divisions in 2 districts	Stillbirths and neonates	136	A) Mother: delivery place; B) Child: 5; 7; 8	Yes	1) Probably not; 2) No	1/2/3) None stated	1/2/3) None stated

de Savigny [17]	2004	Tanzania: Rufiji DSS	Under 5 years old	320 (all malaria)	2; 4a, b, c	Presumed, not demon-strated	1) Yes; 2) No	**□**1/2) Yes; 3) None stated	**□**1) Yes; 2/3) None stated

Bojalil [23]	2007	Mexico: Hidalgo state	Under 5 years old	75 ARI & diarrhea	PtoS study: 3; 4a, c; 5; 6; 7	Yes	1) Yes; 2) Yes	△1/2/3) None stated, but the study aimed to "provide information to better implement interventions linked with IMCI program"	1/2/3) None stated

Beiersmann [18]	2007	Burkina Faso: sub-portion of 1 district	Under-5 years old with malaria	100	4c; 6	Yes	1) Yes; 2) No	1/2/3) None stated	1/2/3) None stated

Waiswa [24]	2010	Uganda: Iganga/Mayuge DSS	Neonates	64	PtoS study: A) Mother: delivery place and attendant; B) Child: 3; 5; 7	Yes	1) No; 2) No	1/2/3) None stated	1/2/3) None stated


Fawcus [39]	1996	Zimbabwe: 1 province and urban Harare	Maternal	166	5; 6; 7; 8; 9	Yes	1) Possible; 2) Yes	12/3) None stated	△1) Yes; 2/3) None stated

Castro [36]	2000	Mexico: 3 states	Maternal	145	3; CS decision maker; 4a, c; 5; 6; 7; 8	Yes	1) Yes; 2) Yes	**□**1) Yes; 2/3) None stated	1/2/3) None stated

Supratikto [38]	2002	Indonesia: 3 districts, S. Kalimantan	Maternal	130	4c, 5; 6; 7	Yes	1) Possible; 2: Yes	**□**1/2/3) Yes	△1) Yes; 2/3) None stated

Bartlett [34]	2005	Afghanistan: Kabul & 3 districts	Maternal	133	4c; 5; 6; 7	No	1) Possible 2) Yes	**□**1) None stated; 2/3) Yes	1/2/3) None stated

Campbell [35]	2005	Egypt	Maternal	718 (1992/3) / 580 (2000)	3; 4c; 5; 7	Yes	1) Yes; 2) Yes	**□**1/2/3) Yes	△1) Passive; 2/3) None stated

UNICEF [10]	2008	India: 4 districts in 3 states	Maternal	102 (1 district)	3; 4a, b, c; 5; 6	Yes	1) Possible; 2) Yes	**□**1/2/3) Yes	**□**1/2) Yes; 3) None stated

Jafarey [37]	2009	Pakistan: 2 districts	Maternal	128	3; 4c; 5; 6; 7; 8	Yes	1) Possible/No; 2) Yes	**□**1) Yes: 2/3) None stated	1/2/3) None stated

D'Ambruoso [40]	2010	Burkina Faso: 1 district; Indonesia: 2 districts	Maternal	70 (BF) / 104 (Indonesia)	5; 6; 7	Yes	1) No; 2) Yes	1/2/3) None stated	1/2/3) None stated

CS: care seeking; HH: household; ARI: acute respiratory infection; PtoS: Pathway to Survival; IMCI: Integrated Management of Childhood Illness; DSS: demographic and surveillance site; △ and **□**: studies ranked, respectively, as providing "any" and "strong" support to health programs and communities.

### Child social autopsy: providing evidence on failures in the Pathway to Survival

The multicountry assessment of WHO/UNICEF's Integrated Management of Childhood Illness (IMCI) approach found that, although IMCI health facilities provided the gold standard of child illness care in developing countries, the strategy failed to decrease child mortality. In part this was due to weak implementation of IMCI's family and community component, and assuming that quality health care services alone would lead to increased care seeking and appropriate home care practices [11]. Access, coverage, and utilization were all found to lag, resulting in ineffective delivery of appropriate child survival technologies.

**Figure 1:**
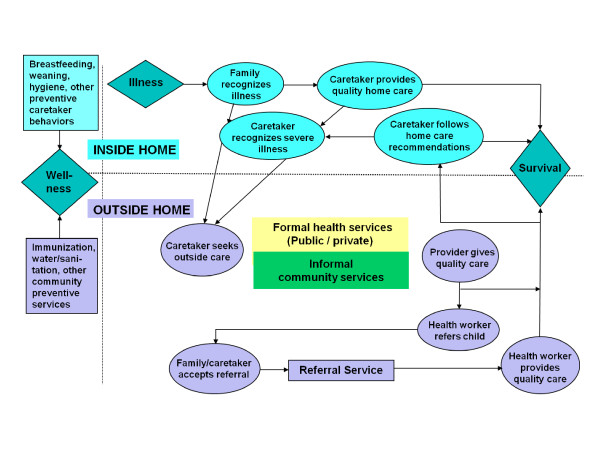
**The Pathway to Survival**.

The Pathway to Survival conceptual framework (Figure 1) was designed to support the implementation and monitoring of IMCI, with the aim of highlighting the essential steps that need to be taken both inside the home and in the community to prevent child illness and return sick children to health. The pathway identifies and organizes modifiable social, cultural, and health system factors affecting home care practices, health care access and utilization, and the delivery of quality health care [12].

As seen in Table 1 (and Additional file 1, which provides more details), the three child social autopsy studies conducted prior to the development of the Pathway model [13-15], as well as the three non-Pathway studies conducted after the Pathway model was developed [16-18], examined, on average, three aspects of the care-seeking process. In contrast, the eight later studies that followed the Pathway model [8,9,19-24] on average assessed eight care-seeking elements, providing a more complete understanding of the care-seeking process and the factors affecting health care utilization for severely ill children in developing countries. Nevertheless, even the non-Pathway studies, with their limited set of data variables and none examining a representative sample of deaths at the district or higher level (two of the Pathway studies met this objective), attempted to form a social diagnosis of mortality determinants. More of the Pathway studies were also conducted by or in support of health programming or health policy development (5/8) and to support community participation and empowerment (4/8), compared, respectively, to 2/6 and 1/6 of the non-Pathway studies. Somewhat higher percentages of the Pathway than non-Pathway studies were also rated as strongly supporting health programs (3/8 versus 2/6) and communities (2/8 versus 1/6).

The first analysis of child deaths following the Pathway model was a survey conducted in 1995 of 271 child deaths from randomly selected census tracts over a then-recent nine-month period in El Alto, Bolivia [8]. The social autopsy format used in the Bolivia study, consisting of a separate sheet duplicating the same open-ended questions on possible problems along the pathway for each day of the illness, was found to be cumbersome both for data collection and analysis. Subsequent work produced a one-page social autopsy tool formatted as a matrix, with each row constituting one action taken for the illness, and columns for recording the action, the illness day the action was taken, the illness signs at the time the action was taken, reasons for the action being taken, and each of the remaining steps along the pathway. The Pathway Analysis social autopsy format was published online as part of a manual aimed at health programs describing how to undertake a child mortality study to determine the biological causes and social determinants of death [25]. Later work added a module for investigating perinatal deaths.

**Figure 2:**
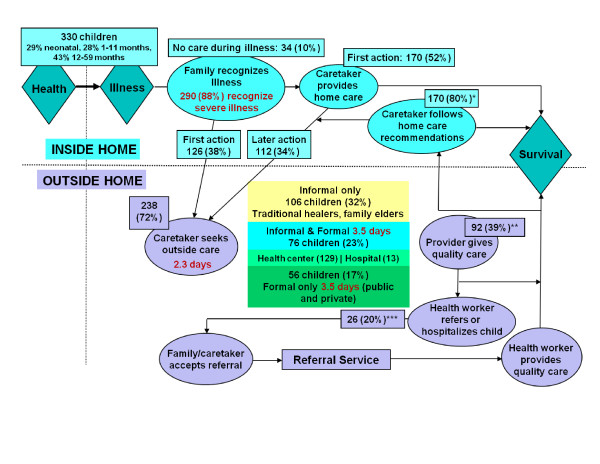
Pathway analysis for 330 child deaths in Mandiana Prefecture, Guinea; denominators: * = 212 children seen by an informal or formal provider and not referred or hospitalized, ** = 238 children seen by a formal or informal provider, *** = 132 children seen by a formal health provider.

Subsequent social autopsy studies utilizing or based on this format and manual were conducted in Guatemala [19], Cambodia [21], and Guinea [9] with support from the group producing the materials, as well as by an independent group working in Uganda [24]. Figure 2 illustrates the type of information gathered by the social autopsy instrument with data from Guinea for 330 child deaths. It can be seen, for example, that while 290 (88%) of the children's caretakers recognized one or more signs of a severe illness, 34 (10%) of the children received no care whatsoever, 238 (72%) were taken for some outside-the-home care, on average 2.3 days after the illness began, and only 132 (40%) children received some formal health care, on average 3.5 days after the illness onset. The Guinea study identified only 13 referrals, perhaps because it used an early version of the social autopsy questionnaire that did not ask about this directly; a later refinement improved the assessment of referrals.

In 2009, the WHO/UNICEF-supported Child Health Epidemiology Reference Group (CHERG) [26] undertook to review and update the Pathway Analysis social autopsy format. The main issues considered were: 1) in response to the increased contribution of neonatal deaths to overall child mortality resulting from recent decreases in post-neonatal deaths [27], to improve the format's assessment of stillbirths and neonatal deaths and related care-seeking issues by adding modules on maternal and newborn care, including care seeking for maternal complications; 2) to strengthen the evaluation of child preventive care; 3) to examine a host of behavioral and social factors not previously considered; and 4) to include questions on the utilization of trained community health workers, in accord with the recent inclusion of these workers in some formal health systems. Most of the social, behavioral, and preventive factors that were added to the questionnaire (Table 2) were based on interventions included in the Lives Saved Tool [28], which undergo rigorous review for evidence of their efficacy. In addition, where possible, the questions were worded similarly to those in the Demographic and Health Surveys (DHS) [29] in order to facilitate comparisons of the social autopsy data with similar data for survivors in settings where a recent DHS was conducted.

**Table 2:** Social, behavioral, and preventive factors included in the updated Pathway Analysis social autopsy questionnaire

Social factors
• Mother's education, literacy, age at marriage • Household possessions, husband's education, breadwinner's occupation • Duration of residence in community and time to reach usual health provider • Social capital (community joint action, helpful persons/groups, denial of services)

Maternal factors (including care seeking for complications)
• Antenatal care (blood pressure, urine and blood, counseling on food and care seeking), tetanus toxoid, insecticide-treated bed net use, malaria prophylaxis • Birthplace and attendant, partograph, handwashing, clean delivery surface • Knowledge of and care seeking for pregnancy, labor, and delivery complications • Constraints to health care seeking and compliance with referral advice for maternal complications • Quality of health care services (treatment, referral, and reasons for referral for complications)

Care seeking for child illnesses
• Newborn and child illness recognition, health care seeking, compliance with treatment, and referral advice • Constraints to health care seeking and compliance with treatment and referral advice • Quality of health care services (treatment, referral, and reasons for referral of sick children).

CHERG integrated the updated social autopsy instrument with the Population Health Metrics Research Consortium verbal autopsy questionnaire, which is currently being extensively validated by studies described in other articles in this series of *Population Health Metrics*. While the updated Pathway Analysis format is considerably longer than the original version, CHERG is developing CAPI (computer-assisted personal interview) software for field-based data capture on a netbook or tablet computer with built-in consistency checks, automatic mapping of skip patterns, and correct question wording depending on who the respondent is and the child's age at death. This should significantly ease the interview process and increase the quality of the data. Software versions are being developed both for the integrated verbal/social autopsy (VASA) interview and for the social autopsy alone to enable using it with other verbal autopsy instruments. These tools will be available open access on the CHERG website in order to facilitate their widespread use.

The largest scale of the prior Pathway Analysis studies was at the provincial level. One last objective that CHERG is working to fulfill is to collaborate with government and international partners in several countries in Africa to develop national and other large-scale VASA studies. The purpose is to provide evidence of modifiable social, cultural, and health systems factors contributing to neonatal and child mortality for advocacy and health policy and planning exercises and to begin gathering the data needed to develop global estimates of these factors.

### Maternal death inquiry and response

Maternal death audit has been undertaken in many forms, including clinical audit, which evaluates the quality of care provided in health facilities against an accepted standard; confidential inquiry of all or a sample of deaths in a population, most often focusing on medical factors but sometimes including community aspects; facility-based maternal death review, preferably augmented with information from the community; and community-based VASA, most useful in areas where many deaths occur outside of a health facility, and which can be combined with a facility review of cases that did access care for a more accurate assessment of medical factors.

From its beginnings with Great Britain's nationwide system of confidential inquiry into maternal deaths, to its promotion by WHO's Beyond the Numbers effort and beyond, several developing countries have implemented a system of maternal death audit. Examples include Sri Lanka [30] and Malaysia [31], which review both hospital and home deaths, and South Africa [32], which conducts confidential inquiries of hospital deaths. The most recent large-scale effort has been the MAPEDIR program undertaken in 10 high-mortality states of India with assistance from UNICEF [10]. Because up to half or more of maternal deaths in these states are thought to go unreported, and many of these are thought to occur at home, it was decided to initiate MAPEDIR with community-based VASA of maternal deaths. In 2010, this effort culminated in the Government of India announcing its plan to commence a nationwide program of facility- and community-based maternal death audits [33].

**Figure 3:**
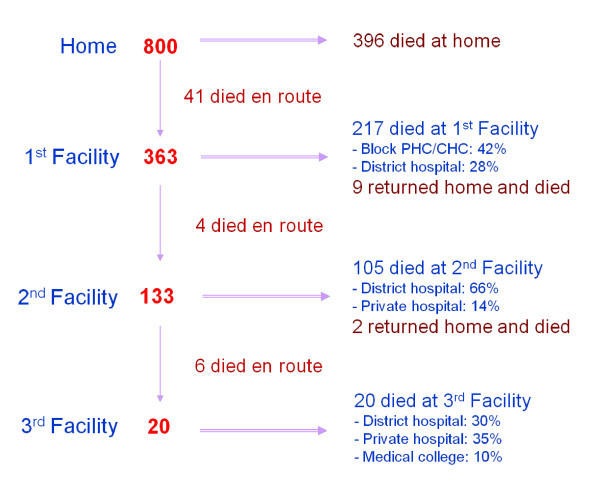
**Pathway analysis for 800 maternal deaths, April 2005 to September 2007, in eight districts of Orissa, India; PHC: primary health care center, CHC: community health center**.

The rationale for maternal social autopsy is the same as for child deaths. There are several highly efficacious interventions against maternal mortality, including, for example, antibiotics for preterm premature rupture of the membranes to prevent maternal (and fetal) sepsis, a skilled birth attendant providing active management of the third stage of labor to prevent postpartum hemorrhage, and treatment of primary postpartum hemorrhage with rectal misoprostol. Yet, as illustrated by the findings of 800 VASA interviews in Orissa, India (Figure 3), many women in developing countries may die at home without ever seeking health care for their maternal complications, and many who do seek care never receive effective treatment. As in the India context, this may often be true despite the fact that multiple facilities at a level that should be capable of providing basic or comprehensive emergency obstetric care are visited during the fatal illness. To effectively tackle these problems, data are needed on the social, behavioral, and health system factors contributing to the deaths. And effective sharing of such data, in a setting where in its absence many of the deaths would not have even been registered much less investigated, can raise awareness of the magnitude, causes, and determinants of the problem and support the development of effective interventions with communities and health programs. Where programs and government are less responsive, the data can be used for advocacy to promote accountability.

Of the seven non-MAPEDIR studies included in the comprehensive review of maternal social autopsy, five strongly supported health programs and three provided some support to community participation and empowerment (Table 1). Two of the studies were either conducted or commissioned by the country's national maternal health program and their findings were used to help guide the country's reproductive health strategy [34,35]. In such cases, it is evident that responsive programs are in place and making good use of the social autopsy data. Two additional papers included an author from the government health authority [36,37], suggesting that practical use might also be made of their findings; one of these stressed the importance of disseminating the findings to policymakers, health planners, health professionals, and the community to ensure sensitization and action and recommended implementation of community- and hospital-based maternal death audits [37]. One other paper described the use of the social autopsy data in a participatory audit system consisting of periodic meetings of community and district health staff, with the audits of some deaths also involving community representatives [38]. The two remaining papers proposed health system or community interventions based on their study findings, but took the process no further [39,40]. Similar to the child studies, all but one of these maternal studies made a social diagnosis of mortality determinants, although only two collected data on a representative sample of deaths from a district or larger area.

The eighth maternal study reviewed, the MAPEDIR program, collected data on a similar number of care-seeking variables (6) as the other seven studies (mean = 4.9), but was unique in its extensive sharing, interpretation, and use of the data for health planning and intervention development by the community, as well as health authorities and government officials (Additional file 1) [10]. The first level of data provided by MAPEDIR was a simple but powerful one in raising awareness and the visibility of the problem. MAPEDIR increased the reporting of maternal deaths by the community and local health providers, most highly in locales where reporting had previously lagged far behind. It accomplished this by first engaging health officials, talking about the problem, and highlighting the reluctance of first-line health workers to report maternal deaths for fear of being blamed and penalized. The importance of a nonblaming approach and a search for systemic causes for the deaths was discussed. Similar sensitization sessions were held with villagers as part of discussions on the need for birth preparedness, complication readiness, and reporting and investigating maternal deaths to discover what went wrong. Data from the resulting death inquiries were sometimes disturbing to health officials and spurred them to take action. For example, in several states it was found that a disproportionate share of maternal deaths occurred among women of lower castes. Data from the inquiries shared with communities empowered them to understand how future deaths could be prevented and to develop appropriate interventions. Table 3 lists some of the many interventions developed at multiple levels as part of the MAPEDIR response.

**Table 3:** Some maternal health interventions undertaken in India in response to MAPEDIR's social autopsy findings

• Dholpur, Rajasthan: taxi union, local NGO, and district health society collaborated in planning and running an obstetric help line and referral transport system
• Guna, Madhya Pradesh: district mapped maternal deaths and revitalized SHC and PHCs in high mortality areas for 24/7 safe-delivery services; district ensured referral transport to all PHCs via call center and secured vehicles (local communities donated 6/22 vehicles)
• Purulia, West Bengal: four gram panchayats (local governance board) initiated and supported van rickshaws intervention for referral transport from isolated villages
• West Bengal: state made all public maternity beds nonpaying; expanded JSY to all SC/ST and BPL women
• Eight Navajyoti districts, Orissa: functional blood banks and blood storage units
• Orissa: state considered how to target men with maternal care-seeking messages

BPL = below poverty line; JSY = Janani Suraksha Yojana (institutional care incentive scheme); MAPEDIR = Maternal and Perinatal Death Inquiry and Response program; NGO = non-governmental organization; PHC = primary health care center; RCH II PIP = Reproductive and Child Health Program phase 2 program implementation plan; SC/ST = scheduled castes and tribes; SHC = subhealth care center.

## Discussion

### Meeting the social autopsy objectives

The comprehensive review of the English and French literature found that adoption of the Pathway to Survival framework, starting with Aguilar et al's 1998 study in Bolivia [8], increased the richness of the data on the care-seeking process collected by child social autopsy. The Pathway studies provided valuable information and an increased understanding of the social, behavioral, and health systems factors affecting care seeking for severe neonatal and child illnesses in developing countries. Thus, while many of the non-Pathway studies made a social diagnosis of mortality determinants, it is likely that the Pathway studies were able to reach a more accurate diagnosis. The Pathway studies also were used somewhat more often to support health programs and communities, but even they fell short of fully achieving this objective -- fewer than half were rated as strongly supportive of health programs and only one-quarter were strongly supportive of communities.

The reviewed maternal social autopsy studies collected data on a range of care-seeking variables, with the MAPEDIR program ranking somewhat above average in the number of factors examined, perhaps enhancing its ability to reach an accurate social diagnosis of mortality determinants. However, while most of the maternal studies strongly supported health programs, only the MAPEDIR program also offered strong support to communities. Recent evidence suggests that this is more than a philosophical choice, that community participation and empowerment can strongly impact neonatal and perhaps maternal mortality [41].

Social autopsy serves varied purposes; among these are providing large-scale population-level data to contribute to country and global estimates of mortality determinants, and increasing awareness of maternal and child death as preventable problems in order to empower communities and engage health programs. While most of the reviewed studies did not meet the objective of selecting a representative sample of deaths at the district or larger area level, and so may not be generalizable to overall mortality in the study areas and thereby serve the first listed purpose of social autopsy, nonetheless several were found to strongly support health programs and communities in improving health interventions and access to care. However, this does not negate the importance of selecting cases in as representative a fashion as possible, within existing limitations. For example, if it is known that many deaths in the service area are occurring at home, then a social autopsy investigation should be designed to ensure that home deaths are included in the study sample.

### The relationship of social autopsy to death audit

The term "social autopsy" implies that a social diagnosis is to be made of the most common or otherwise important social, behavioral, and health systems determinants of mortality. While data on social factors can play a role in audits of individual deaths, as in facility-based death reviews that include interviews of the deceased's family members, the term as used in this review refers to aggregate diagnoses made using quantitative data. Social autopsy can also provide qualitative data in the form of individual illness narratives, with the purpose of illustrating the overall care-seeking process, showing how several problems can conspire to cause a death, and putting a human face on the numbers. Sharing and discussion of these narratives, especially at the community level, provides a powerful learning opportunity, but the process must take care to preserve confidentiality and foster a nonblaming approach. Otherwise, there is the danger that the sharing session could deteriorate into a negative atmosphere, diverting attention from the necessary focus on systemic problems that can be fixed through collective action and discouraging community participation.

Supratikto et al. conducted community-based audits of local, individual maternal deaths, and discussed the challenge of preserving confidentiality and nonblaming in this setting [38]. In their research study of three different approaches to village-based audit of neonatal deaths, Patel et al. did not face this problem, though they observed that the bereaved family's presence at the audit could decrease participants' willingness to discuss the case, and cautioned that in other settings scrutinizing individual cases might adversely affect family and community relations [42]. The MAPEDIR program's approach to maintaining confidentiality while utilizing illness narratives was to share composite vignettes of cases illustrating common problems or actual cases from across borders so the participants would be unlikely to know the family of the case being discussed. In the end, the discussion would be brought back to the quantitative data to help prioritize problems illustrated by the individual stories within the context of the community's overall experience.

### Limitations

Just as in the sharing of social autopsy data with the community, care must be taken in collecting and handling the data to ensure confidentiality of the highest possible level and the respondent's comfort with providing the information. There is potential for the information to be stigmatizing, for example, if the respondent or another family member delayed taking an action perceived as possibly life-saving. This could affect the respondent's openness during the interview and the accuracy of the illness reports. Limited recall for remote events is another potential problem, as sample size needs for child social autopsy may require interviewing families with a child death three or more years ago. This issue will be more exacerbated in a study of maternal deaths.

### Technical issues

As with verbal autopsy, some challenges remain for social autopsy, both to improve its ease of use and assess the validity of the information it gathers. Item reduction might be possible, both in terms of the number of questions asked and the number of potential responses to multiple-choice questions. This would shorten the duration of the interview and simplify the questions, making it more practical to conduct social autopsy on the platform of national surveys and in the context of local health program planning and monitoring. An integrated VASA interview can take 60 to 90 minutes to complete, and some have argued that the interviews should therefore be separated. Counterarguments are that respondents would rather not undergo a second visit; that a return visit would be even more impractical on the platform of a national survey; that elements of the verbal autopsy, such as the illness signs and symptoms, must in any case be discussed as part of the social autopsy care-seeking questions; and that by following a more natural chronology an integrated interview promotes an improved interviewer-respondent dialogue and enhances recall of the illness events. The new CAPI software also promises to ease the interview process and shorten its duration.

Another argument in favor of separating the verbal and social autopsies is that this might help overcome the potential for the stigma of the social autopsy discussed above to affect disclosure of verbal autopsy information and hence determination of the biological diagnoses. This separation could also foster a specialized approach to social autopsy, enabling a more elaborate informed consent process and a dedicated cadre of interviewers trained to more effectively deal with feelings of anger, guilt, and shame over the death that might arise during the interview. Determining which of the implementation models works best will require additional experience and possibly experimentation.

Social autopsy analysis at its most basic level involves outputting frequency distributions of all the questionnaire's mortality determinants. However, many important questions can be answered only by cross tabulations, such as who the decision-makers were for facility and home delivery, and for which illness signs and symptoms particular actions were taken. Comparisons can also be made of women or children for whom care was and was not sought. This can enhance the identification of care-seeking constraining factors. Such analyses might also help with item reduction by identifying noninformative variables, and potentially expose needed areas of inquiry not covered by the questionnaire.

While there is a history of validating verbal autopsy, only one study included in the comprehensive review validated any aspect of the social autopsy. Bojalil et al. independently assessed the clinical competence of doctors mentioned in mothers' narratives of their child's fatal illness and found a high correlation between the quality of care they provided as assessed from the narratives and by the competence scores [23]. Jafarey et al. attempted to use medical records to validate third delays identified in narrative accounts of care received for maternal complications at tertiary facilities, but were not able to due to inadequate information in the medical records [37]. In addition to the quality of medical care received, other aspects of social autopsy to consider for validation include caregivers' reports of care sought or other actions taken, and care-seeking delays and constraining factors.

### The way forward

It is apparent that the Pathway to Survival and Three Delays models are useful for organizing the care-seeking process for severe child and maternal illnesses. Future social autopsy studies should be guided by these models, and should also make use of social autopsy's other strengths of raising awareness through participatory data sharing and intervention development. Just as there have been international efforts to standardize verbal autopsy instruments and analytic methods for neonatal, child, and adult deaths, the utility and acceptability of social autopsy findings could be advanced by reaching agreement on a core dataset to be gathered and on standardized formats and methods for accomplishing this. CHERG's Pathway Analysis format has the advantage of having been reviewed and updated by scientists from several international organizations, including WHO, but the team was small, the instrument has not been officially endorsed, and there are other groups working to develop their own social autopsy instrument for child deaths, the most prominent being the INDEPTH Network. A process is needed to bring together interested parties, finalize these efforts, and reach agreement on a standardized format and data analysis plan.

Similarly, a standardized social autopsy format and analysis plan for maternal deaths is needed. WHO published suggested questions for maternal verbal autopsy, including questions on care seeking [43], that at least two of the papers in the present review used as the basis for their study instruments. UNICEF's MAPEDIR program developed a standardized VASA format for investigating maternal deaths as well as accompanying materials for training surveyors and sharing the findings with the community, but has not yet published these resources.

Developing standardized questionnaires and analysis plans for child and maternal deaths could promote the agenda of routinely collecting and utilizing quality social autopsy data. These instruments should be provided together with tools for country adaptation, fieldworker training, and community data sharing. These efforts will be enhanced by further work to develop the social autopsy method, such as identifying ways to minimize the stigmatizing effect of certain questions and assessing the optimal model for combining or sequencing the verbal and social autopsy interviews.

## Conclusions

Social autopsy is a powerful tool for raising awareness and the visibility of child and maternal death as preventable problems in the community, among health workers, health authorities, and government officials. It provides evidence in the form of actionable data to communities, health programs, and health policymakers, and increases motivation at all levels to take appropriate and effective actions. Health systems and communities of implementing countries, individually and as partners, used the Pathway to Survival and MAPEDIR findings to develop appropriately focused interventions. Additional social autopsy studies reviewed for this paper were utilized by governments to improve health programming. Social autopsy data can also build institutional awareness and political commitment, thus helping to increase health system and governmental accountability and responsiveness. Community participation in the death inquiry and response process may in itself act as an intervention by increasing awareness and motivating communities to take action in a way that increases care seeking. Social autopsy studies conducted in representative, large-scale populations can provide data to develop national and global estimates of social, cultural, and health systems determinants of mortality and to advocate for the resources needed to overcome these problems. New neonatal and child VASA studies are being planned at the country or subnational level in several African countries. Further development of the social autopsy method and development and wide-scale adoption of standardized tools based on the Pathway to Survival and Three Delays models will promote social autopsy's overall objectives of providing evidence on failures in the pathway to survival and increasing awareness to empower communities and engage health programs in the battle against child and maternal mortality.

## Competing interests

The authors declare that they have no competing interests.

## Authors' contributions

HDK co-led the team that developed the Pathway Analysis questionnaire, led the teams that updated this instrument and developed the MAPEDIR questionnaire, participated in the design and conduct of the Pathway Analysis study in Guinea and the MAPEDIR program in India, drafted the manuscript, and co-conducted the comprehensive review of social autopsy studies. RS participated in the design and conduct of the Bolivia Pathway Analysis study, co-led the team that developed the Pathway Analysis questionnaire, and was a member of the team that updated the instrument. MB conceived of the MAPEDIR program in India and led UNICEF monitoring and technical assistance to the government. AK helped draft the methods section of the manuscript and co-conducted the comprehensive review of social autopsy studies. REB conceived of updating the Pathway Analysis questionnaire. All authors critically reviewed and approved the final manuscript.

## Supplementary Material

Additional file 1 **Studies and reports meeting the inclusion criteria of the comprehensive review (with detailed findings)**. CS: care seeking; SA: social autopsy; HH: household; ARI: acute respiratory infection; MOH: ministry of health; PHC: primary health care; AD: acute diarrhea; VA: verbal autopsy; DSS: demographic and surveillance site; PtoS: Pathway to Survival; SB: stillbirth; DHS: Demographic and Health Survey; CHW: community health worker; VHC: village health committee; ANC: antenatal care; IMCI: Integrated Management of Childhood Illness; MOHP: ministry of health and planning; GOWB: Government of West Bengal; NGO: non-governmental organization; PRI: panchayat raj institutions; **□ **and **□**: studies ranked, respectively, as providing "any" and "strong" support to health programs and communities. Click here for file
